# Household-level data on well-being, inequalities, and social capital in Western Province, Zambia

**DOI:** 10.1016/j.dib.2024.110504

**Published:** 2024-05-15

**Authors:** Martin Schlossarek, Jaromír Harmáček, Lenka Dušková, Lenka Suchá

**Affiliations:** a Palacký University Olomouc, Department of Development & Environmental Studies, 17. listopadu 12, 771 46 Olomouc, Czechia; b Global Change Research Institute of the Czech Academy of Sciences, Bělidla 986/4a, 603 00 Brno, Czechia

**Keywords:** Human development, Intra-household inequalities, Deprivations, Decision-making power, Aspirations, government perceptions

## Abstract

This article presents survey data from households from the Muoyo-Mukukutu area in Western Province, Zambia based on stratified sampling. Data from 411 households were collected using a questionnaire survey from 2022. Understanding the complexities of well-being is crucial for informing policies to enhance the quality of life and reduce multidimensional poverty in developing countries. Hence, the survey focuses on subjective and objective well-being and their determinants. Survey data contains details on various dimensions of objective well-being, such as living standards, health, and nutrition. It also covers the issue of subjective well-being (life satisfaction), including the related concept of freedom of choice. Moreover, we collected detailed information about diverse forms of inequalities and deprivations at the societal and intra-household level, paying particular attention to the areas of social capital and decision-making power. Additionally, the data contain details about the relationships with and attitudes to traditional leaders and statutory government representatives, respondents’ economic activities and aspirations (with a special focus on agriculture), and their various socio-demographic characteristics. Individual survey results can be compared with a robust set of data as we intentionally used questions applied in other international surveys when possible.

Specifications Table

**Table:** 

Subject	Social Sciences
Specific subject area	Planning and Development, Sociology, Political Science, Anthropology
Data format	Raw data
Type of data	dta file, xlsx file
Data collection	The questionnaire survey took place from 18 to 28 July 2022. We used stratified sampling. Question items are based on qualitative research previously conducted in the area, and on various sources, most of them backed by respected international organizations such as the World Bank or UN agencies (see below).
Data source location	Data were collected in the Muoyo-Mukukutu area, Western Province, Zambia, by the team led by researchers from Global Change Research Institute and Palacký University Olomouc, Czech Republic.
Data accessibility	Repository name: Mendeley Data Data identification number: https://doi.org/10.17632/xkg94kmcjs.1 Direct URL to data: https://data.mendeley.com/datasets/xkg94kmcjs/1 Instructions for accessing these data: [1]

## 1 Value of the Data

• We provide representative regional data exploring subjective and objective well-being, emphasizing determinants including living standards, health, and freedom of choice. The data also delve into societal and intra-household inequalities, focusing on social capital, decision-making power, and attitudes towards traditional leaders (as representatives of customary governance system) and statutory government representatives. Additionally, they examine economic activities, aspirations, and socio-demographic characteristics.
• The data enable the examination of subjective and objective well-being as well as influencing factors (e.g., social capital, decision-making powers, intra-household inequalities, and economic aspirations).
• The data also allow for an exploration of respondents’ perceptions of government trustfulness and inclusiveness, as well as differences in perceptions between traditional leaders and statutory government representatives.
• The data bring a broad range of value to development cooperation practitioners, policymakers, and researchers to study entry points for (multidimensional) human development.
• The detailed data collected can be used as a standalone database for comparison with data from other regions and in meta-analyses. While the data are collected in a relatively small area in Zambia's Western Province, they can reflect a more general experience of poor farming households in some remote and disadvantaged agricultural areas of Sub-Saharan Africa.

## 2 Background

The importance of subjective well-being (SWB) in policymaking has been increasing [2,3]. While SWB has been extensively studied in the Global North, the Global South, specifically Sub-Saharan Africa, has received less attention even though this topic is relevant for designing policies on equal opportunities, human development, and poverty alleviation. SWB is, for example, determined by objective well-being and/or human development dimensions (living standards, health, education; [[4], [5], [6]], socio-demographic characteristics such as age [7,8], and economic and non-economic inequalities [9,10]).

## 3 Data Description

**Fig. 1:**
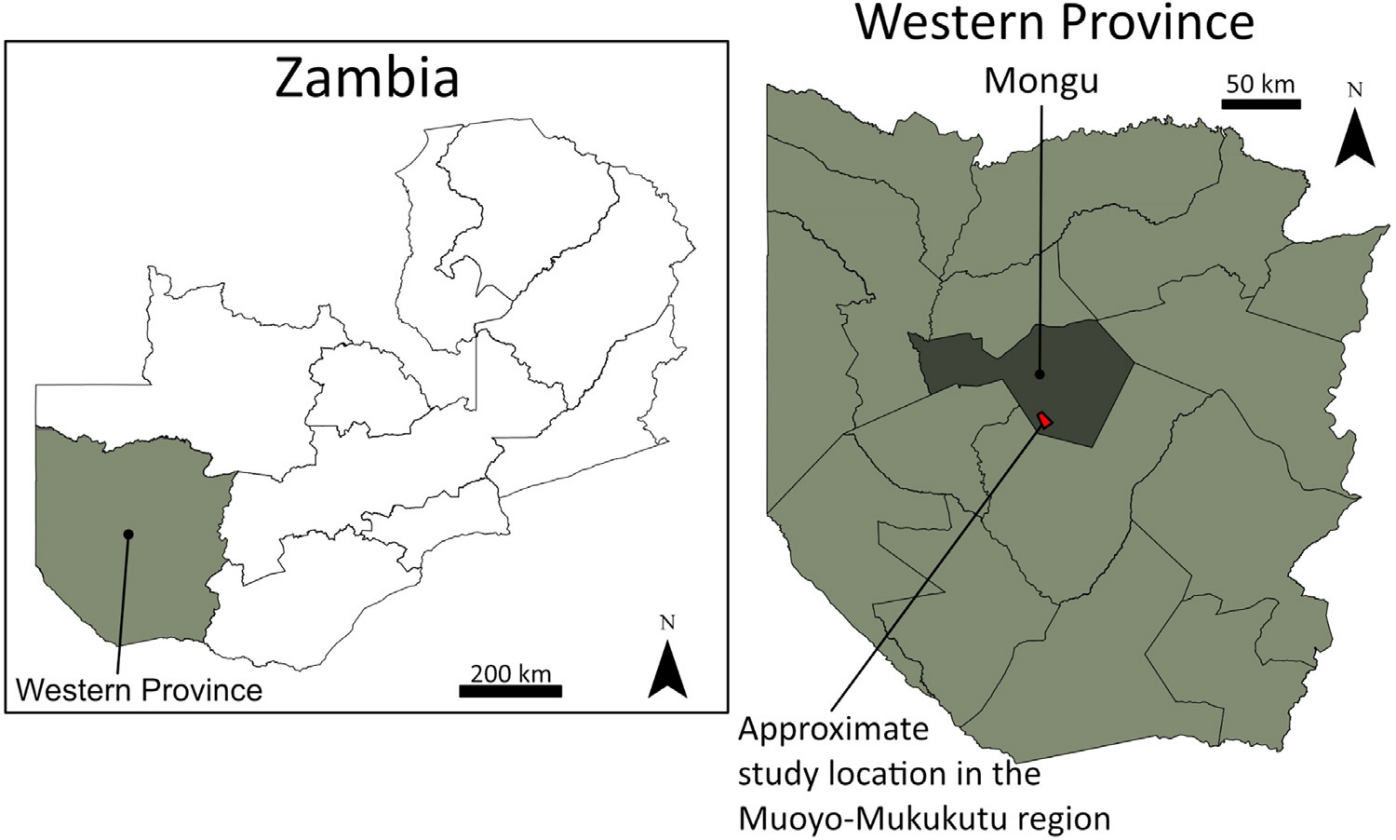
Location of the survey area.

Presented data are based on the questionnaire survey conducted in Muoyo town and Mukukutu village south of Mongu, which is the capital of Zambia's Western Province (see Fig. 1). Muoyo was selected to represent a typical regional township located close to the strategic M10 road, surrounded by a cluster of socio-economically and demographically similar settlements. Mukukutu represents a rural settlement with a significant percentage of small farmers, low transport accessibility, and underdeveloped infrastructure. It is located on the edge of a Zambezi floodplain, which is extensively used for irrigation-intensive crops such as rice. We collected 411 responses from the inhabitants of the area. The data collection took place from July 18 to July 28, 2022.

The overarching focus of the questionnaire survey was on the well-being of local residents. More specifically, the data collection aimed at obtaining detailed information about subjective and objective well-being and an extensive set of individual and household characteristics. We covered in detail the objective well-being of households and individuals via the list of assets and housing characteristics, health and nutrition information, and education. On an individual level, we also focused on respondents’ control over their life choices and various dimensions of social capital and attitudes to/trust in the statutory government representatives and traditional leaders. Except for the last one, all previous items are expected to be relevant factors of subjective well-being. Attitudes/trust are added rather as an exploratory set of variables, bearing value per se, as questions focused on government representatives and traditional leaders delve into the topic of perceptions of “traditional” versus “modern” leadership. In total, the survey contains 150 questions, but some of them are conditional (depending on previous answers). Our survey data can be compared with data from other sources since we intentionally used questions applied in international surveys (when it was possible and appropriate).

The survey area is disadvantaged compared to the Zambian average in multiple aspects. For example, reported life satisfaction in Muoyo-Mukutu is 3.05, while the national score is 3.98 (on a scale from 1 to 10, 10 being the best; [11]). In Mukutu-Muoyo, only 64 % of people are satisfied with the freedom of choice they have in their life, compared to 79 % nationally [11]. People in the area are also poorer, as seen in Table 1. We highlight these differences between national statistics and our Muoyo-Mukukutu data to emphasize that researchers should be careful when generalizing their findings based on our dataset.

**Table 1:** Comparison of ownership rates of selected assets with Zambian national data.

Possession	Muoyo – Mukukutu	National average
radio	27.6 %	47.1 %
refrigerator	4.9 %	21.9 %
television	11.0 %	36.6 %
mobile phone	73.7 %	73.7 %
bicycle	6.8 %	37.8 %

The dataset (including codebook describing the variables), and questionnaire are available on the Mendeley Data: https://data.mendeley.com/datasets/xkg94kmcjs/1.

## 4 Experimental Design, Materials and Methods

Stratified sampling was done by dividing each of the two municipalities (Muoyo, Mukukutu) into four geographically defined areas. Muoyo was partitioned into four quadrants: North-West, South-West, North-East, and South-East. The town's main road (the local branch of M10) delineates the demarcation between the northern and southern quadrants. In the southern part of the town, the boundary between the western and eastern quadrants is demarcated by an unnamed road originating from the main junction situated 100 m northeast of the secondary school. In the northern part of the town, the boundary between the western and eastern quadrants is defined by an imaginary straight line perpendicular to the local branch of the M10 road at the aforementioned junction. Mukukutu was partitioned based on the official administrational neighbourhoods: Mukukutu Central, Mantondo, Kandiyana, and Mushukula.

Next, quotas were set for the representation of age categories, sex, and household heads (self-identified in the questionnaire; versus ordinary household members) for data collection purposes so that the structure of the resulting sample roughly matched available data (age, sex) or estimates (heads of household) from the local government. Furthermore, the data enumerators were instructed to create the sample with qualities similar to those derived from a stratified random sampling procedure.

The questionnaire pilot was followed by an intensive training of five data enumerators and then by the actual collection of the data. During data collection, we conducted two assessments to ascertain adherence to quotas: first after reaching 200 questionnaires, and second after reaching 300 questionnaires. In both cases, observations revealed that age categories and sex were within the designated parameters. However, the representation of heads of households exceeded the predetermined range. Subsequently, we instructed our data collectors that within the household, *they* should be the ones choosing respondents (and not the household members, who usually tend to nominate a head of household to represent them). Since we were close to reaching the quotas for two of the criteria (age and sex), and because the data collectors repeatedly confirmed they were following all instructions for the data collection carefully, we opted *against* implementing more stringent measures to decrease the share of the household heads in our sample.^1^

After finishing data collection, we found that, except for the heads of households, the quotas were respected. However, in this context it needs to be noted that the representative share of household heads for the Muoyo-Mukukutu area is a bit vague. This depends on two factors: the number of adults in a household, and (perhaps surprisingly) the number of people who claim they are head of household. Assuming that there is always *exactly one* person in each household who claims to be its head, our sample should have approximately 47 % of household heads. There are not many studies focusing on the topic of head of household being claimed by more household members, but for example, in a South African qualitative study dealing with intra-household relationships among 30 households, three households (i.e., 10 %) had a dispute over the household head role [12]. The higher the share of such households where more people believe they are heads of households, the higher the percentage of heads of households correctly found by our enumerators. We believe this effect can explain part of the difference between the quota (47 %) and the result (67 %), see Table 2. Nevertheless, the other part may be attributed to a sampling bias, potentially arising from factors such as the disproportionate availability of heads of households compared to other household members during the data collection period.

**Table 2:** Quotas for the sample.

Quota based on:	Quotas – details	Result
Age	10 to 14 % of people being 60+ years old	10.2 %
Sex	43 to 48 % of women	43.1 %
Head of household	40 % to 60 % of heads of households	67.0 %

During data collection, the average response time was around 45 min. The raw data that accompanies this article is structured around the design of the survey, which consisted of seven thematic areas:

(i) Socio-economic and demographic information about the individuals and the household
(ii) Indicators of subjective well-being
(iii) Indicators of objective well-being
(iv) Intra-household interactions and social capital
(v) Governance perceptions
(vi) Farming and related topics

The seventh topical area, *Inequality and deprivations*, was cross-sectional.

Questions related to a specific area are not necessarily grouped together in one section of the questionnaire, in fact, they are often scattered in its various parts (see Appendix B for the questionnaire).

### 4.1 Socio-economic and demographic information about the individuals and the household

In the first section, basic information about the respondent is collected. We obtain information about where the respondent lives (Muoyo or Mukukutu and which one of the four geographically defined areas in each locality) as well as basic socio-demographic details such as sex, age, marital status (married, widowed, divorced, never married), and native language^2^ (Lozi versus other language, which is specified in the follow-up open question). We also focus on the household structure: we ask who the head of the household is, what their education is, and what the number of household members is (including the number of adults and children).

### 4.2 Indicators of subjective well-being

In the second section, we ask the respondents two questions about life satisfaction. While the first concerns the current state, the second one is future oriented and asks about the predicted life satisfaction in 5 years time from the perspective of the respondent. The first question is widely used as an indicator of subjective well-being. Both questions use a 1 to 10 scale, 1 being the “worst possible life” for the respondent, and 10 being the “best possible life.” We also ask about satisfaction with the freedom to choose what to do with their life (answers were only “yes” or “no”). In this section, we worked with questions assessing the quality of life used in the Gallup World Poll [11]. Data for these questions are collected at the country level annually in more than a hundred countries worldwide.

### 4.3 Indicators of objective well-being

This section of the questionnaire is based on the three elementary dimensions of human development (HD) as reported, for example, in the Human Development Index [13]. These are living standards, health, and education. Regarding the living standards, we include questions focused on the quality of housing (such as the type of roofing and material used for walls), and assets owned by the household (such as radio, refrigerator, and bicycle).^3^ The living standards questions are identical to the ones used in the last Zambian Demographic and Health survey [14] or Multidimensional Poverty Index Questionnaire, version 2020.1 [15]. As for asset ownership, we ask a follow-up question whether the item is owned by the respondent, jointly owned by the respondent and somebody else, or owned by another household member. This allows for measuring intra-household inequality in access to resources.

A series of questions measure health: respondents self-evaluate various dimensions of their health, such as pain, immunity, outlook, and general health (all of them use a 5-point Likert scale). These questions are taken from the Rand 36-Item Health Survey [16]. We also ask whether an under-18-year-old household member died in the last five years (this question comes from [15]).

We also focus on nutrition, which is closely associated with the state of health in poor communities. We ask about the lack of food, and intra-household inequality associated with food access. As for the lack of food, we are interested in: (1) whether it happened that household members ate less than they wanted due to lack of resources over the last 12 months, and (2) whether it happened that some household members did not eat at all for a whole day due to lack of resources over the last 12 months. In case of household inequality in food access, we want to know whether it is true that some other person in the household eats more or better meals than the respondent, and if it is the case, then we ask whether the respondent perceives it as fair or not.

In terms of education, the third dimension of human development, we ask about: (1) respondents’ highest attained education, and (2) the education attainment of the most and least educated adult persons in the household. This allows us to analyze intra-household education distribution and inequality. For households that have one or more children at the age of compulsory schooling, we ask whether these children attend school.

### 4.4 Intra-household interactions and social capital

In this section we cover intra-household power relations and ask questions to approximate the respondent's social capital. We focus on the following dimensions of social capital: informal relations, formal relations, and the existence of contacts that can arrange a valued service for the respondent.

Questions focused on power relations are based on hypothetical scenarios, which we prepared after conducting qualitative research in the area. We ask whether different situations would lead to the respondent being forced to leave the house: divorce, death of a spouse, death of somebody else (responses are on a 4-point Likert scale, from “definitely yes” to “definitely no”). The other four questions are based on scenarios of a dispute between household members regarding moving out of the village, and crop cultivation (5-point context-specific Likert scale). The variable of interest is the perceived ability of the respondents to assert their will.

As for the (formal) social capital, we first ask about group membership (farming groups, saving groups, etc.), and whether the respondent is in a leadership position in any group. We then delve into the issue of group(s) members diversity in terms of education, occupation, and place of residence. While the first question measures the “quantity” of membership, questions on leadership position and diversity serve as proxies for “quality”, or “how beneficial” the group membership is.

Then, questions about (informal) social capital follow. We want to know how many friends the respondents have, and what their socio-demographic diversity is, aiming again at measuring the “quantity” and “quality” of the phenomena. Finally, we ask whether the respondents have friends who can provide a valuable service for them, specifically a money loan or borrowing a car (5-point Likert scale, from “definitely” to “definitely not”). Most of the questions related to social capital are taken from or inspired by the World Bank´s Integrated questionnaire *Measuring Social Capital* [18].

### 4.5 Governance perceptions

In this section, we first focus on respondents’ trust in various government representatives (statutory government representatives, i.e., members of the national parliament, executive government officers, and judges, as well as traditional leaders including Litunga [the king of the Lozi people], area chiefs, senior headmen, and village headmen). We ask how honest they are in the opinion of the respondent (on a 5-point Likert scale from “very dishonest” to “very honest”).

Then we focus on the interactions between respondents and the government representatives. We ask whether respondents feel they have a higher/same/lower chance of “getting assistance” from them (one by one for the same representatives as above, except that we skip the question on members of the national parliament). Then we are interested whether these representatives (including a member of the national parliament) know them personally. While questions focused on honesty are inspired by [17], the rest is inspired by our qualitative research done in the area prior to the quantitative data collection.

A series of general questions about equality in access to assistance from the government representatives follow. Here we distinguish only statutory government representatives versus traditional leaders, and we focus on differences between “poor people” versus the rest, and “some people” versus the rest. Responses are recorded on a 5-point Likert scale.

The last part of this section deals with decision-making processes in the community. Four decision-making processes are described, from the most inclusive (“People hold a discussion and decide together”) to the least inclusive (“The decision is imposed from outside”). Respondents answer on a 5-point Likert scale how often such decision-making processes happen in their community.

### 4.6 Farming and related topics

The final section is focused on farming and related topics. First, we ask about the respondents’ income-generating activities’ structure (what they do for living, and what is the most important source of their income). We distinguish four categories: crop farming, animal farming, (non-agricultural) employees with a salary, and (non-agricultural) businesspeople.

Then we delve into crop farming, the most important economic activity in the region. We ask what crops the respondents plant. If they cultivate rice (the most important crop in the area), we are interested in the size of their rice fields, and in the last yield. Also, we want to know whether they plant rice in lines, which is a rough proxy of how farmers stick to the advice of the local agricultural extension officer. Questions about working aspirations follow. We ask about the respondents’ dream job: they choose between working in or outside agriculture, and then between being self-employed/entrepreneur versus being an employee. Based on this series of questions, we can identify (mis)match between what respondents *do* and what they *would like to do*.

Finally, we focus on the issue of land tenure. In two separate questions, we ask how confident the respondent is that the statutory government representatives, or traditional leaders, are able to protect the respondent's land against an illegal use by somebody else. Responses are recorded on a 4-point Likert scale, from “very confident” to “not confident at all”. The last two questions are about land disputes. Respondents react on a 4-point Likert scale (from “strongly agree” to “strongly disagree”) to the following two statements: “In this village/town, people do not trust each other in matters of land ownership”, and “In this area, problems with land disputes and acquisition have increased over the last five years.”

### 4.7 Inequalities and deprivations

Inequalities and deprivations are cross-sectional topic, and we have already indicated in previous sections how we measure inequalities and deprivations based on the data we have. We focus on various dimensions of intra-household distributions, and on social capital. Regarding the former one, it is possible to measure distribution of access to resources, nutrition, education, and decision-making. In the case of social capital, it is possible to measure distribution of its various dimensions, such as group membership (formal social capital), friendships (informal social-capital), and trust in and contacts with the local government and traditional leaders (governance-based social capital).

## Limitations

We assert/argue that the Muoyo-Mukukutu area exhibits common socioeconomic characteristics, including constrained access to resources, infrastructure, and markets, when compared to other parts of Zambia, and Sub-Saharan Africa. Moreover, agricultural practices typified by small-scale subsistence farming and technological limitations are prevalent in many other parts of the region. The same applies to development challenges such as restricted access to education, healthcare, and basic services. Consequently, the observed patterns and dynamics in the data are likely reflective of *some* other regions of Zambia and Sub-Saharan Africa.

However, the data are collected in a relatively small area in Zambia's Western Province. Therefore, our results may be subject to influences such as local habits, traditions, or environmental nuances not even fully discerned by researchers. For that reason, users should take caution in generalizing the results either to the Western Province, to Zambia, or even to the region of Sub-Saharan Africa. Additionally, we suspect that the data are biased towards a higher share of heads of households. This issue was discussed in the Experimental Design, Materials, and Methods section.

## Ethics Statement

The informed consent was read to each respondent before they filled out the questionnaire. The consent is available from the authors on request. The data were collected in a larger research project that follows the Ethical Codex of the Global Change Research Institute of the Czech Academy of Sciences (Regulation No. 1/2017).

## CRediT author Statement

**Conceptualization:** Schlossarek, Harmáček, Suchá, Dušková; **Methodology:** Schlossarek, Harmáček; **Validation:** Schlossarek, Harmáček; **Investigation:** Schlossarek, Harmáček; **Formal analysis:** Harmáček, Schlossarek; **Writing – Original Draft:** Schlossarek, Harmáček; Writing – Review & **Editing:** Schlossarek, Harmáček, Suchá, Dušková; **Project administration:** Schlossarek, Suchá; **Funding acquisition:** Suchá.

## Data Availability

Dataset on Subjective and Objective Well-being from the Muoyo-Mukukutu Area in Zambia's Western Province (Original data) (Mendeley Data)

## Appendix Supplementary materials

Image, application 1

## Footnotes

1 One measure under consideration was instructing the data collectors to first inquire within the household about the head of the household and then include another adult member in the survey.

2 This question can also serve as a proxy for ethnic groups and potentially for discrimination as Lozi people have vast majority in the area.

3 Based on these variables, it is possible to create an asset index measuring material well-being. See, for example, [15].
