# Supplementary material for: Household-level data on well-being, inequalities, and social capital in Western Province, Zambia
Source: Data Brief. 2024 May 15;54:110504. doi: 10.1016/j.dib.2024.110504 (PMC11144721; doi:10.1016/j.dib.2024.110504)
Supplement: Supplementary file 1 [file mmc1.pdf]

TACR questionnaire wellbeing

This research aims to analyze wellbeing of people in the area, specifically satisfaction with social relations and governance. The information obtained by the research can potentially help local NGOs to help people more effectively. The questionnaire has 4 parts. In the first section, basic information about you will be collected. In the second section, we will ask you about your household wellbeing. In the third and fourth part, we will ask you about social relations and governance. Please, feel free not to answer questions that are not comfortable for you. Remember that there are no good and bad or right and wrong answers. The questionnaire will take approximately up to 40 minutes to complete. Be aware that all data you provide are confidential and will be used only for research purposes. Answers in the questionnaires will never be made public by name and they will never influence your participation in any future projects organized by government, NGO, or any other stakeholder.

---

1 **DO NOT READ THE QUESTION: Where does the respondent stay?**

- ◯ In Muoyo
- ◯ In Mukukutu

2 **DO NOT READ THE QUESTION: Which part of Muoyo?**

- ◯ Southeast
- ◯ Northeast
- ◯ Southwest
- ◯ Northwest

3 **DO NOT READ THE QUESTION: Which part of Mukukutu?**

- ◯ Mukukutu - main part
- ◯ Mantondo
- ◯ Kandiyana
- ◯ Mushukula

4 **DO NOT READ THE QUESTION: What is the sex of respondent?**

- ◯ male
- ◯ female

5 **What is your age?**

____

6 **Are you married?**

- ◯ Yes
- ◯ No

7 **Have you ever been married?**

- Yes
- No

8 **Are you divorced or widowed?**

- Divorced
- Widowed

9 **What is your native language?**

- Lozi
- Other

10 **What language is it?**

____

11 **What is the highest level of school you attended?**

- No education
- Primary school
- Junior secondary school
- Higher secondary school
- College
- University

12 **What is the highest grade you completed at that level?**

____

13 **During the past 4 weeks, how much did pain interfere with your normal work (including both work outside the home and housework)?**

- Not at all
- A little bit
- Moderately
- Quite a bit
- Extremely

**Do you agree with the following statements?**	Strongly disagree	Disagree	Neither agree nor disagree	Agree	Strongly agree
14 **I seem to get sick a little easier than other people.**	○	○	○	○	○

15 **My health is excellent**	◯	◯	◯	◯	◯
16 **I expect my health to get worse**	◯	◯	◯	◯	◯

17 **Are you (personally) satisfied or dissatisfied with your freedom to choose what you do with your life?**

- ◯ Satisfied
- ◯ Dissatisfied

18 **Please imagine a ladder with steps numbered from zero at the bottom to 10 at the top. The top of the ladder represents the best possible life for you and the bottom of the ladder represents the worst possible life for you. On which step of the ladder would you say you personally feel you stand nowadays?**

____

19 **On which step do you think you will stand about five years from now?**

____

20 **Whom do you consider as the head of your household?**

Do not read the list.

- ◯ Me
- ◯ Husband/wife
- ◯ My father
- ◯ My mother
- ◯ Someone else (male)
- ◯ Someone else (female)

21 **What is the age of the head of household?**

____

22 **What is the highest level of school attended by the head of household?**

- ◯ No education
- ◯ Primary school
- ◯ Junior secondary school
- ◯ Higher secondary school
- ◯ College
- ◯ University
- ◯ I do not know

23 **Including yourself, how many people currently live in your household?**

____

24 **How many of them are adults? By adults, we mean people who are 18 and older.**

____

25 **How many of them are children? By children, we mean all people younger than 18.**

Check whether children plus adults make same total as hinted in previous question about the total number of people in household.

Please confirm that total number of people in this household is Q14 = Q15 plus Q16

____

26 **Please report the information about: house flooring**

Observe main material and decide (do no read the list)

- ◯ Earth/sand
- ◯ Cement (concrete)
- ◯ Dung
- ◯ Wood planks
- ◯ Palm/bamboo
- ◯ Finished floor
- ◯ Parquet or polished wood
- ◯ Vinyl or asphalt strips
- ◯ Ceramic tiles
- ◯ Carpet
- ◯ Other

27 **If other, please indicate which type.**

____

28 **Please report the information about: house walls**

Observe main material and decide (do no read the list)

- Bricks
- Dirt (mud, dung wall)
- Cane/palm/trunks (thatched walls)
- Cement blocks
- No walls
- Bamboo with mud
- Stone with mud
- Uncovered adobe
- Plywood
- Cardboard
- Reused wood
- Cement
- Stone with lime/cement
- Covered adobe
- Wood planks/shingles
- Other

29 **If other, please indicate which type.**

____

30 **Please report the information about: house roofing**

Observe main material and decide (do no read the list)

- ○ Thatch/palm/leaf
- ○ Metal (iron sheets)
- ○ No roof
- ○ Sod
- ○ Rustic mat
- ○ Palm/bamboo
- ○ Wooden planks
- ○ Cardboard
- ○ Wood
- ○ Calamine/cement fiber
- ○ Ceramic tiles
- ○ Roofing shingles
- ○ Other

31 **If other, please indicate the type.**

____

32 **Please report the information about: Sanitation**

Ask the question: „What kind of toilet facility do members of your household usually use?" and decide (do no read the list)

- ○ Pit latrine without slab / open pit
- ○ Pit latrine with slab
- ○ Ventilated Improved Pit latrine
- ○ No facilities or bush or field
- ○ Flush to piped sewer system
- ○ Flush to septic tank
- ○ Flush to pit (latrine)
- ○ Flush to somewhere else
- ○ Flush to unknown place
- ○ Composting toilet
- ○ Bucket
- ○ Hanging toilet/hanging latrine
- ○ Other

33 **If other, please indicate the type.**

---

34 **Do you share this toilet facility with other households?**

If they say NO, then ask: "Is it correct that nobody else than those X members of the household uses the toilet regularly?"

- Yes
- No

35 **Please report the information about: cooking fuel**

Ask the question: „What type of fuel does your household mainly use for cooking?" and decide (do no read the list)

- Charcoal
- Wood
- Electricity
- Liquid Propane Gas (LPG)
- Natural gas
- Biogas
- Kerosene
- Coal / Lignite
- Straw/shrubs/grass
- Agricultural crop
- Animal dung
- No food cooked in household
- Other

36 **If other, please indicate the type.**

---

37 **Please report the information about: primary source of drinking water**

Ask the question: „What is the main source of drinking water for the household members?" and decide (do no read the list)

- Unprotected well
- Protected well
- Tubewell/borehole
- Surface water (river, stream, dam, lake, pond, canal, irrigation channel)
- Water piped into dwelling
- Water piped into yard or plot
- Public tap/standpipe
- Water from protected spring
- Water from unprotected spring
- Rainwater
- Tanker-truck
- Cart with small tank/drum
- Bottled water

38 **Please report the information about: primary source of non-drinking water**

Ask the question: „What is the main source of water used by your household for other purposes such as cooking and handwashing?" and decide (do no read the list)

- Unprotected well
- Protected well
- Tubewell/borehole
- Surface water (river, stream, dam, lake, pond, canal, irrigation channel)
- Water piped into dwelling
- Water piped into yard or plot
- Public tap/standpipe
- Water from protected spring
- Water from unprotected spring
- Rainwater
- Tanker-truck
- Cart with small tank/drum

39 **How long does it take to get to the water source, get water and come back?**

- Water on premises
- Minutes

40 **If minutes, how many?**

____

41 **Does your household have electricity?**

Answer yes if it is connected to the grid or if solar panel is there & electricity is main source of lighting

- ◯ Yes
- ◯ No

42 **Does your household have a radio?**

To answer yes, it cannot be broken.

- ◯ Yes
- ◯ No

43 **Does your household have a refrigerator?**

To answer yes, it cannot be broken.

- ◯ Yes
- ◯ No

44 **Does your household have a television?**

To answer yes, it cannot be broken.

- ◯ Yes
- ◯ No

45 **Does your household have a mobile phone?**

To answer yes, it cannot be broken.

- ◯ Yes
- ◯ No

46 **Do you personally own it?**

- ◯ Yes, I own it
- ◯ Yes, but it is shared ownership
- ◯ No, it is owned by someone else

47 **Does your household have a bicycle?**

To answer yes, it cannot be broken.

- ◯ Yes
- ◯ No

48 **Do you personally own it?**

- Yes, I own it
- Yes, but it is shared ownership
- No, it is owned by someone else

49 **Does your household have a motorbike/scooter?**

To answer yes, it cannot be broken.

- Yes
- No

50 **Do you personally own it?**

- Yes, I own it
- Yes, but it is shared ownership
- No, it is owned by someone else

51 **Does your household have a car?**

To answer yes, it cannot be broken.

- Yes
- No

52 **Do you personally own it?**

- Yes, I own it
- Yes, but it is shared ownership
- No, it is owned by someone else

53 **Does your household have a truck?**

To answer yes, it cannot be broken.

- Yes
- No

54 **Do you personally own it?**

- Yes, I own it
- Yes, but it is shared ownership
- No, it is owned by someone else

55 **Does your household have a computer/laptop?**

To answer yes, it cannot be broken.

- Yes
- No

56 **Do you personally own it?**

- Yes, I own it
- Yes, but it is shared ownership
- No, it is owned by someone else

57 **Does your household have an animal-drawn cart?**

To answer yes, it cannot be broken.

- Yes
- No

58 **Do you personally own it?**

- Yes, I own it
- Yes, but it is shared ownership
- No, it is owned by someone else

59 **Please answer the following questions about nutrition in your household. During the last 12 MONTHS, was there a time when some members of this household ate less than you thought they should because of a lack of money or other resources?**

- Yes
- No

60 **During the last 12 MONTHS, was there a time when some people in this household went without eating for a whole day because of a lack of money or other resources?**

- Yes
- No

61 **Is it true that some other adult(s) in this household eat(s) more or better meals than you?**

- Yes
- No

62 **Do you think it is fair that some other adult(s) in this household eat(s) more or better meals than you?**

- Yes
- No

63 **Did anybody of age below 18 die in this household in the last five years?**

If they say NO, then ask: "Any baby who cried or showed signs of life but did not survive?"

If they say no -> ask: Any baby who cried or showed signs of life but did not survive?

- Yes
- No

64 **What is the highest level of school attended by the MOST educated person in the house?**

- No education
- Primary school
- Junior secondary school
- Higher secondary school
- College
- University
- I do not know.

65 **What is the highest grade the most educated person completed at that level?**

____

66 **What is the highest level of school attended by the LEAST educated person in the house? Take into account only people above the age of 18.**

- No education
- Primary school
- Junior secondary school
- Higher secondary school
- College
- University
- I do not know.

67 **What is the highest grade the least educated person completed at that level?**

____

68 **Are there children of age between 6 to 12 in your household?**

- Yes
- No

69 **Did all children of age between 6 to 12 attend school or pre-school at any time during the 2021-2022 school year?**

- Yes, all of them
- No, only some of them
- No, none of them
- We do not have children of that age in the household

70 **Suppose you and your spouse were to get divorced. Do you think you would be forced to leave this house?**

- Definitely yes
- Probably yes
- Probably no
- Definitely no
- Refused to answer

71 **And supposed – and we apologize as we know this may be hard to think about – your spouse was to pass away. Do you think you would be forced to leave this house?**

- Definitely yes
- Probably yes
- Probably no
- Definitely no
- Refused to answer

72 **Suppose – and we apologize as we know this may be hard to think about – somebody else in the household was to pass away. Do you think you would be forced to leave this house?**

- Definitely yes
- Probably yes
- Probably no
- Definitely no
- Refused to answer

73 **Imagine the following scenario. You want to move with your family to another village/town, but the most influential person from your household disagrees. What will happen?**

The most influential person can possibly be the spouse, the head of household, somebody else.

- We will move there
- Probably we will move there
- I am not sure
- Probably we will not move there
- We will not move there

74 **Imagine the following scenario. The most influential person in your household wants to move with your family to another village/town, but you disagree. What will happen?**

The most influential person can possibly be the spouse, the head of household, somebody else.

- We will move there
- Probably we will move there
- I am not sure
- Probably we will not move there
- We will not move there

75 **Imagine the following scenario. You want to start growing other crop than you are used to in your household, but the most influential person in your household disagrees. What will happen?**

The most influential person can possibly be the spouse, the head of household, somebody else.

- We will grow it
- Probably we will grow it
- I am not sure
- Probably we will not grow it
- We will not grow it

76 **Imagine the following scenario. The most influential person wants to start growing other crop than you are used to in your household, but you disagree. What will happen?**

The most influential person can possibly be the spouse, the head of household, somebody else.

- We will grow it
- Probably we will grow it
- I am not sure
- Probably we will not grow it
- We will not grow it

**In your opinion, how honest are the following officials? Please rate them on a 1 to 5 scale, where 1 is very dishonest and 5 is very honest.**	1 - very dishonest	2	3	4	5 - very honest
77 **Members of the parliament**	○	○	○	○	○
78 **Executive government officials (people from local ministries, extension officers)**	○	○	○	○	○
79 **Judiciary officials (judges)**	○	○	○	○	○
80 **Litunga**	○	○	○	○	○
81 **Your area chief**	○	○	○	○	○
82 **Your senior headman (induna)**	○	○	○	○	○
83 **Your village headman**	○	○	○	○	○

84 **Do you think you have the same chance to get assistance from your village headman as other people?**

- Yes
- No

85 **Is the chance higher or lower?**

- Higher
- Lower

86 **Do you think you have the same chance to get assistance from your senior headman (induna) as other people?**

- Yes
- No

87 **Is the chance higher or lower?**

- Higher
- Lower

88 **Do you think you have the same chance to get assistance from your area chief as other people?**

- Yes
- No

89 **Is the chance higher or lower?**

- Higher
- Lower

90 **Do you think you have the same chance to get assistance from Litunga as other people?**

- Yes
- No

91 **Is the chance higher or lower?**

- Higher
- Lower

92 **Do you think you would be treated equally as other people by judges?**

- Yes
- No

93 **Is the chance higher or lower?**

- Higher
- Lower

94 **Do you think you have the same chance to get assistance from executive government officers (extension officers, local ministries) as other people?**

- Yes
- No

95 **Is the chance higher or lower?**

- Higher
- Lower

96 **Does your village headman know you personally?**

- Yes
- No

97 **Does your senior headman (induna) know you personally?**

- Yes
- No

98 **Does your area chief know you personally?**

- Yes
- No

99 **Does Litunga know you personally?**

- Yes
- No

100 **Is there a judge who knows you personally?**

- Yes
- No

101 **Is there a member of parliament who knows you personally?**

- Yes
- No

102 **Is there an executive government official (extension officer or somebody from local ministries) who knows you personally?**

- Yes
- No

	Do you agree with the following statements?	Strongly disagree	Disagree	Neither agree nor disagree	Agree	Strongly agree
103	**Poor people have a lower chance to get assistance from the official government representatives.**	◯	◯	◯	◯	◯
104	**Some people have a higher chance to get assistance from the official government representatives.**	◯	◯	◯	◯	◯
105	**Poor people have a lower chance to get assistance from traditional leadership.**	◯	◯	◯	◯	◯
106	**Some people have a higher chance to get assistance from the traditional leadership.**	◯	◯	◯	◯	◯

	When there is a decision to be made in your village/town, how often the following happens?	It (almost) never happens	It happens rarely	It happens sometimes	It happens often	It happens (almost) always
107	**People hold a discussion and decide together.**	◯	◯	◯	◯	◯
108	**The village headman decides and informs the other group members.**	◯	◯	◯	◯	◯
109	**The village headman asks people what they think and then decides.**	◯	◯	◯	◯	◯
110	**The decision is imposed from outside.**	◯	◯	◯	◯	◯

111 **Give an example when the decision was imposed from outside in your village/town.**

	Are you a member of any informal or formal group(s) from the following list? *NOTE: just attending the church does not count as a membership in religious group.*	Yes, I am a member	No, I am not a member
112	**Women-only group**	◯	◯
113	**Registered farming group**	◯	◯
114	**Non-registered farming group**	◯	◯
115	**Business group**	◯	◯
116	**Savings group**	◯	◯

117 **Group focused on preservation of culture**	○	○

118 **Are you member of any other group not mentioned in the previous series of questions?**

- ○ Yes
- ○ No

119 **What is the focus of this "other" group?**

____

120 **DO NOT READ THE QUESTION: Did the respondent indicate membership in at least one group?**

- ○ Yes
- ○ No

121 **Are you in a leadership position of any such group (chairman, secretary, etc.)?**

- ○ Yes
- ○ No

122 **Do members of the groups in which you are a member mostly have the same occupation as you?**

- ○ Yes
- ○ No

123 **Do members of the groups in which you are a member mostly have the same educational level as you?**

- ○ Yes
- ○ No

124 **Do members of the groups in which you are a member mostly live in the same village/town as you do?**

- ○ Yes
- ○ No

125 **How many close friends do you (approximately) have these days? These are people you feel at ease with, can talk to about private matters, or call on for help.**

____

**Thinking about your close friends, are some of them different from you in terms of...** *Two different denominations of church count as a different religion.*	Yes	No
126 **religion?**	○	○
127 **gender?**	○	○
128 **ethnic background?**	○	○

129 **Do all your close friends have the same occupation as you?**

- ◯ Yes
- ◯ No

130 **Do all your close friends have the same education level as you?**

- ◯ Yes
- ◯ No

131 **If you suddenly needed to borrow a small amount of money (2000 kwacha), are there people beyond your immediate household and close relatives to whom you could turn and who would be willing and able to provide this money?**

- ◯ Definitely
- ◯ Probably
- ◯ Unsure
- ◯ Probably not
- ◯ Definitely not

132 **If you needed to borrow a car for a half-day trip (with a driver if you do not have a license), are there people beyond your immediate household and close relatives to whom you could turn and who would be willing and able to provide you with the car?**

TO BE DECIDED DURING THE PILOT: MOTORBIKE, OX-CART

- ◯ Definitely
- ◯ Probably
- ◯ Unsure
- ◯ Probably not
- ◯ Definitely not

What income generating activities do you have?

133 ☐ Crop farmer
134 ☐ Animal farmer
135 ☐ Employee with a salary
136 ☐ Businessperson

137 **Which one is your most important income generating activity?**

If more than one tick in the previous question!

- ◯ Crop farmer
- ◯ Animal farmer
- ◯ Employee with a salary
- ◯ Business person

What are the most important crops you plant?

Depending on the answer, you can choose from one up to three crops.

138 ☐ rice

139 ☐ maize

140 ☐ cassava

141 ☐ other crop

142 **What crop is the "other" crop?**

____

143 **Last season, did you plant rice in lines?**

Yes if more than a half of the field planted in lines.

- ○ Yes
- ○ No

144 **Last season, how many limas, acres or hectares did you plant with rice?**

____

145 **DO NOT READ THIS QUESTION: Did respondent answer the previous question in limas, acres or in hectares?**

- ○ limas
- ○ hectares
- ○ acres

146 **Last season, how many bags of rice you harvested?**

____

147 **Last season, did you plant maize in lines?**

Yes if more than a half of the field planted in lines.

- ○ Yes
- ○ No

148 **Last season, did you plant cassava in lines?**

Yes if more than a half of the field planted in lines.

- ○ Yes
- ○ No

149 **What fits better to your case?**

- ○ My dream is to work in agriculture.
- ○ My dream is to work outside agriculture.

150 **What fits better to your case?**

- My dream is to have my own big and successful farm.
- My dream is to work as a well-paid employee on a farm.

151 **What fits better to your case?**

- My dream is to built my own succesful business.
- My dream is to work as a well-paid employee in some company.

152 **How confident are you that the TRADITIONAL LEADERS (village headman, senior headman/induna, area chief, litunga) will protect you if somebody tries to use any of your land against your will?**

- Not confident at all
- Not confident
- Somewhat confident
- Very confident
- I do not know

153 **How confident are you that the STATE AUTHORITIES LEADERS (ministry of land / justice/judiciary – court of law) will protect you if somebody tries to use any of your land against your will?**

- Not confident at all
- Not confident
- Somewhat confident
- Very confident
- I do not know

154 **Do you agree with following statement? In this village/town, people do not trust each other in matters of land ownership.**

- Strongly agree
- Rather agree
- Rather disagree
- Strongly disagree
- I do not know

155 **Do you agree with following statement? In this area, problems with land disputes and acquisiton have increased over the last five years.**

- Strongly agree
- Rather agree
- Rather disagree
- Strongly disagree
- I do not know

Thank you very much for your time!
